# OffRisk: a docker image for annotating CRISPR off-target sites in the human genome

**DOI:** 10.1093/bioadv/vbad138

**Published:** 2023-10-06

**Authors:** Gil-ad Barkai, Tal Malul, Yossi Eliaz, Eran Eyal, Isana Veksler-Lublinsky

**Affiliations:** Department of Software & Information Systems Engineering, Faculty of Engineering, Ben-Gurion University of the Negev, Beer-Sheva, Israel; Department of Software & Information Systems Engineering, Faculty of Engineering, Ben-Gurion University of the Negev, Beer-Sheva, Israel; Computer Science Department, HIT Holon Institute of Technology, Holon, Israel; Center for Theoretical Biological Physics, Rice University, Houston, TX, USA; NRGene LTD, Rehovot, Isarel; Evogene LTD, Rehovot, Isarel; Department of Software & Information Systems Engineering, Faculty of Engineering, Ben-Gurion University of the Negev, Beer-Sheva, Israel

## Abstract

**Summary:**

The CRISPR-Cas9 system has been adapted to achieve targeted genome editing as well as transcriptional control by customizing 20-nt guide RNA (gRNA) molecules for desired regions in the target genome. Designing gRNAs must consider nonspecific and unintended binding, known as off-targets, since these may have potentially harmful effects. To assist in gRNA design, we have developed OffRisk. This Docker-based tool annotates off-target sites in the human genome and assigns them a potential risk label by incorporating functional and regulatory information at different molecular levels.

**Availability and implementation:**

OffRisk is available at https://github.com/IsanaVekslerLublinsky/OffRisk and https://github.com/IsanaVekslerLublinsky/OffRisk-ui (including code, user guide, docker installation guide, and running examples).

All processed datasets are available at https://zenodo.org/record/8289271.

## 1 Introduction

Typically, Clustered, Regularly Interspaced Short Palindromic Repeats (CRISPR) in conjunction with a CRISPR-associated nuclease (Cas9) protect bacteria from foreign viral DNA. The system has been adapted to achieve targeted genome editing at non-bacterial genomes simply by designing 20-nt guide RNA (gRNA) molecules against desired regions in the target genome and utilizing the DNA repair machinery of the host. The genome editing process consists of three stages: (i) RNA-guided Cas9 nuclease searches for DNA sequence complementary to the gRNA containing a specific protospacer-adjacent motif (PAM), (ii) Cas9 generates a double-strand break in DNA, and (iii) endogenous DNA repair machinery repairs the lesion (Wang and Doudna 2023). Several species, including monkeys, humans, mice, rats, zebrafish, worms, and flies, have been successfully targeted with CRISPR-Cas9. The striking success of CRISPR gene editing across the tree of life holds promise in a variety of fields, including animal disease modeling, material science, genetically modified plants, biofuels, gene therapy, and drug development.

There are many guidelines to increase the editing success rate when designing a gRNA, such as the requirement of PAM and appropriate GC content. An important issue in gRNA design is the presence of undesirable off-targets. An off-target refers to a genomic location where the Cas9-gRNA complex might cleave, but this location is not the intended target for the gRNA. Off-targeting is a primary concern when using the CRISPR-Cas9 system in clinical and biomedical applications, as unintended off-target edits may cause potentially harmful effects like genomic instability and disrupt gene function, for instance, by activating oncogenes.

Computational tools such as Cas-OFFinder (Bae *et al.* 2014) and FlashFry (McKenna and Shendure 2018) were developed to search for off-target sites of a specific gRNA sequence. Several specificity scores, such as CFD and MIT, were developed to calculate potential off-target activity (Liu *et al.* 2020). Some web-based platforms (e.g. Hodgkins *et al.* 2015, Concordet and Haeussler 2018, Clement *et al.* 2019) take into consideration off-target activity and provide basic information about their location. CRISPOR (Concordet and Haeussler 2018) provides the genomic position of off-target sites and annotation of whether they fall into an exon, intron, or between genes and the closest gene. Similarly, Wellcome Trust Sanger Institute Genome Editing (WGE) database (Hodgkins *et al.* 2015), provides for each off-target site the number of mismatches and the type of site (i.e. intronic, exonic, or intergenic). CRISPResso (Clement *et al.* 2019) is a suite of computational tools to qualitatively and quantitatively evaluate the outcomes of genome-editing experiments (e.g. HDR or NHEJ), including at off-target sites. CRISPRitz (Cancellieri *et al.* 2020) is a software package that provides several functionalities such as off-target site search that supports mismatches and bulges, and annotation of the off-target hits using a set of predefined genomic annotations, e.g. exons, introns, promoters, CTCF and DNase I regions on the genome, or using custom annotations provided by the user. Although all mentioned tools are highly useful, none of the tools provide comprehensive annotations of the targeted genomic features, particularly their connection to diseases.

In this work, we developed OffRisk, a docker-based tool to assist in gRNA design for the human genome. Our tool annotates off-target sites and assigns them a potential risk label by incorporating functional and regulatory information at different molecular levels (i.e. DNA, RNA, and protein). The tool is operated via an easy-to-use and straightforward user interface (UI), which can be run locally on a personal computer as well as on a remote server, providing visual reports that can be explored and downloaded for further use.

## 2 OffRisk overview

### 2.1 Input

OffRisk analyzes and visualizes various information layers on off-target sites in the human genome and evaluates their potential risk. The tool can be run by two input modes: (i) gRNA input: given a gRNA sequence, three programs are offered to search for off-target sites, FlashFry, Cas-OFFinder, or CRISPRitz (parameters of the search, e.g. the number of mismatches and bulges, are configurable); or (ii) Off-targets input: a list of off-target sites that the user supplies.

### 2.2 Analysis

**Figure 1.**
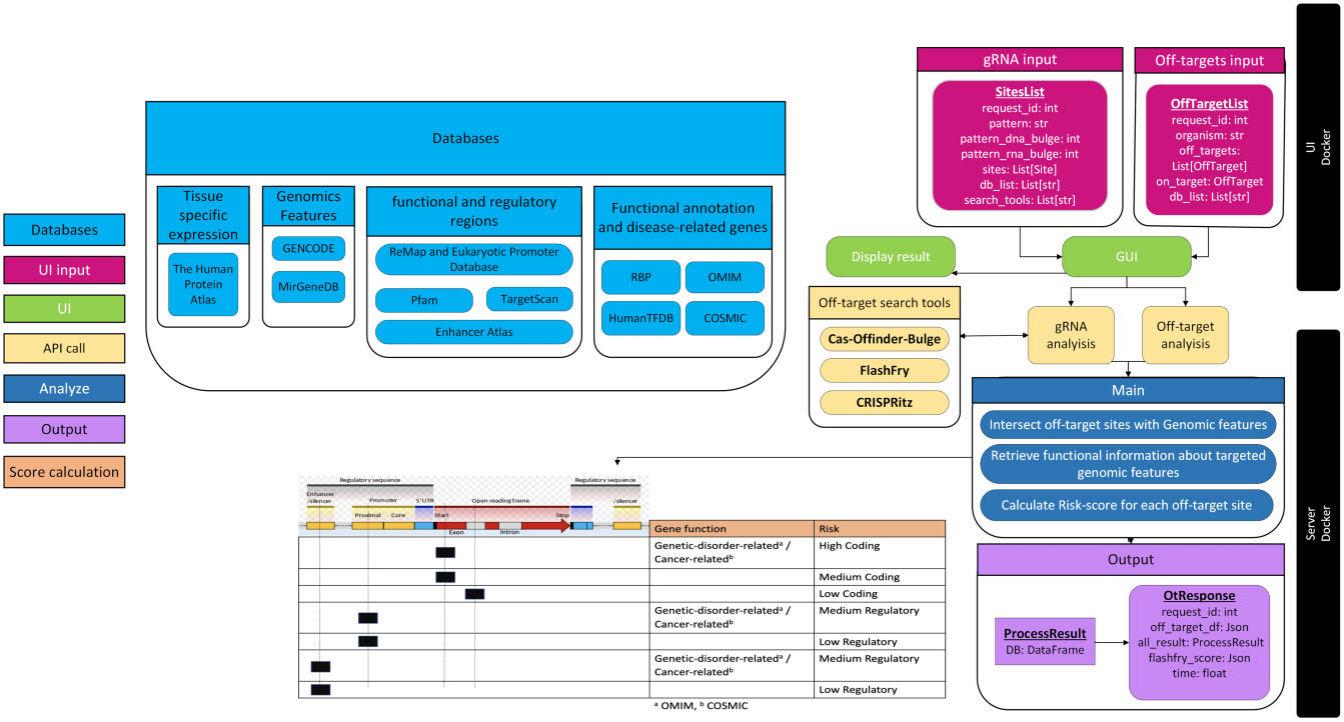
OffRisk structure, including risk label assignment schema. Gene structure illustration was adapted from Shafee and Lowe (2017).

The list of off-target sites is then analyzed against different databases chosen by the user. Briefly, “BEDTools intersect” (Quinlan and Hall 2010) is used to identify genomic features that overlap with off-target sites using genomic locations provided in resources, e.g. GENCODE (gene features), MirGeneDB (miRNA genes), ReMap (transcriptional regulators peaks) with EPD (gene promoter regions), Enhancer Atlas (enhancer regions), Pfam (protein domains) and TargetScan (miRNA target sites). Then, resources, e.g. OMIM (genetic phenotypes), HumanTFDB (transcription factors and co-factors), The Human Protein Atlas (expression profiles in tissues), RNA-binding proteins (RBP functions), and COSMIC (cancer-related genes), are used to retrieve functional annotations of these features using a common identifier (e.g. Gene Ensembl ID). A detailed description of all databases (including references), and of the pre-processing and analysis steps involved in each one of them is provided in the Supplementary Material. Our pipeline also assigns a risk label for each off-target site based on the genomic feature or region it hits (coding/non-coding/regulatory) and the function associated with the feature (schema provided in Fig. 1).

### 2.3 Output

Results are displayed to the user in a UI via tables and charts (see example below). The results for each database are shown in a different table. In addition, one or more columns from these tables are added to a summary table that provides complete information for each off-target site. Importantly, each off-target in the final table is color-coded by the risk label, to allow quick inspection of the results. Charts display off-target locations in the genome, the distribution of risk labels across all off-targets, the distribution of the targeted gene types, and the expression profile of targeted protein-coding genes. All results can be exported in csv or excel format for further analysis.

## 3 Implementation

OffRisk comprises two components, UI and a server; both are implemented as dockers. The UI allows users to send an API request to the server for data analysis. The server returns the computed information to UI in a JSON format to allow easy pragmatical consuming of the results, which is then presented to the user with tables and charts. As OffRisk was built using docker infrastructure, it can be deployed on every architecture supported by the Docker engine (https://www.docker.com/). Docker infrastructure allows seamless download and installation of all the pipeline dependencies and databases. Both dockers are implemented using Python 3.7.9. The UI is implemented with the Streamlit module and the server is implemented with the Flask module. Moreover, along with this tool, we provide the collection of all processed datasets and all pre-processing scripts that can be invoked to update the data used in the analysis (see Supplementary Materials).

## 4 Running example

**Figure 2.**
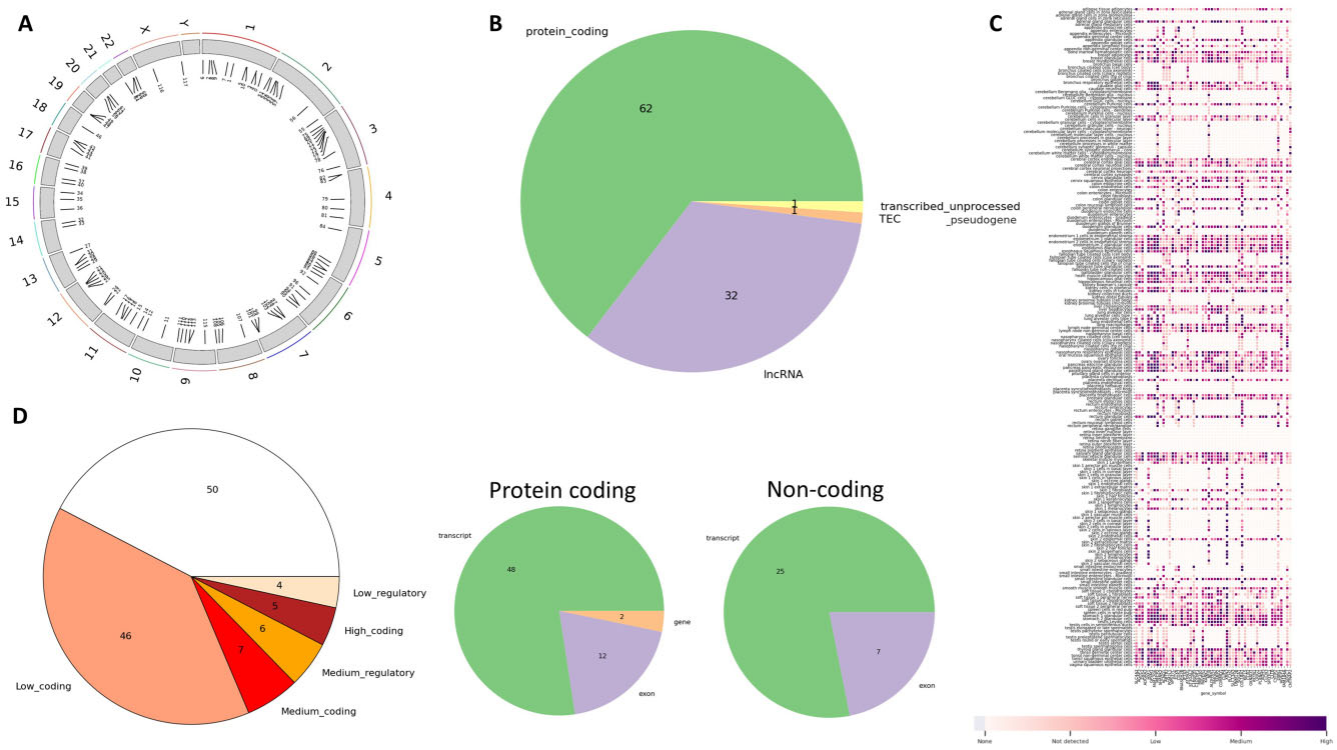
Charts produced by OffRisk when annotating gRNA *CACCCGATCCACTGGGGAGC* that targets the CCR5 gene [taken from Cancellieri *et al*. (2020)]. (A) The genomic locations of the identified 118 sites on the human genome. (B) The distribution of the targeted gene types, including segment types for coding and non-coding genes. (C) Expression profile of targeted protein-coding genes. (D) The distribution of risk labels across all target sites.

To demonstrate the utility of our tool, we ran it on the gRNA *CACCCGATCCACTGGGGAGC* targeting the gene CCR5, which is a therapeutic target for patients with human immunodeficiency virus (HIV) infection. This gRNA was examined in a previous study by the CRISPRitz tool (Cancellieri *et al.* 2020). We ran OffRisk using the *gRNA input* configuration, using CRISPRitz as an off-target search tool, allowing four mismatches. We received a total of 118 sites (Fig. 2A), such that 1-0-1-15-101 sites have 0-1-2-3-4 mismatches, respectively. The site with 0 mismatches corresponds to the on-target site in the CCR5 gene; the rest, 117 represent off-target sites. These target sites fall within 62 protein-coding genes (including CCR5), 32 lncRNAs, one transcribed-unprocessed-pseudogene, and one TEC. Twelve and seven coding genes (including CCR5) and lncRNAs, respectively, are targeted within exons (Fig. 2B). The expression profile of the targeted protein-coding genes within different tissues can be observed in Fig. 2C.

Additional information can be retrieved from a summary table produced by OffRisk: thirteen genes are targeted in their promoter region; fourteen of the sites fall in enhancer regions; one of the exonic sites is inside a protein domain. As for functional information, 10 and 3 sites fall within genes that encode TFs and TF co-factors, respectively; 3 sites hit an RNA binding protein, and 1 of them has a role in RNA processing, stability and decay, and viral RNA regulation; 18 targeted genes are associated with genetic phenotypes, of which 4 are targeted in their exons, including CCR5; three of the targeted genes are cancer-related, of which one is targeted in its exon. None of the off-targets hit miRNA genes or their target sites. Based on all information, a risk label is assigned to each site (Fig. 2D). Five of the sites that fall within exons of protein-coding genes with known genetic phenotypes and/or roles in cancer are classified as *high-coding*, while the rest of the sites within coding genes are either *medium-coding* or *low-coding*. The site on targeted gene CCR5 is the only site with 0 mismatches and is one of the *high-coding* hits. Twenty-five of the sites fall in enhancer or promoter regions, ten of which do not overlap the transcribed area of any protein-coding gene; six of these sites are in regulatory region of genes that have known genetic phenotypes and/or roles in cancer, thus are classified as *medium-regulatory*, and the remaining four are classified as *low-regulatory*. The full HTML report for this gRNA and additional examples can be found in the GitHub repository.

## 5 Discussion and conclusion

With the increasing use of CRISPR technology in the genetic manipulation of human cells, particularly for clinical use, considering the potential effects of off-target editing is crucial. OffRisk framework allows elaborated analysis that goes beyond the computational score of cleavage efficiency by incorporating a number of highly important resources, especially those connecting genes to diseases, and aggregating this information to suggest a risk for each off-target site.

While currently, OffRisk provides annotation only for the human genome, the tool’s architecture is inherently designed to support robust scalability and seamless modularity. Thus, natural future extensions to this tool will include supporting additional organisms and incorporating additional resources and cell-type-specific considerations. Importantly, although OffRisk was originally designed to extract information on CRISPR off-target locations, it can also be used to assist other applications that require analyzing a given set of genomic sites against the resources supported by this tool.

## Supplementary Material

vbad138_Supplementary_Data
