# Supplementary material for: OffRisk: a docker image for annotating CRISPR off-target sites in the human genome
Source: Bioinform Adv. 2023 Oct 6;3(1):vbad138. doi: 10.1093/bioadv/vbad138 (PMC10568243; doi:10.1093/bioadv/vbad138)
Supplement: vbad138_Supplementary_Data [file vbad138_supplementary_data.pdf]

OffRisk: A docker image for annotating CRISPR off-target sites in the human genome

Supplementary material

Gil-ad Barkai, Tal Malul, Yossi Eliaz, Eran Eyal and Isana Veksler-Lublinsky

1 Design and implementation

1.1 Design

As depicted in Figure 1 the pipeline is composed of two components: user interface (UI) and server, which are implemented as dockers: the web UI allows users to send an API request to the server for the data analysis, which returns the computed information back to UI in a JSON format. This information is then presented to the user with tables and graphs, depending on the chosen database.

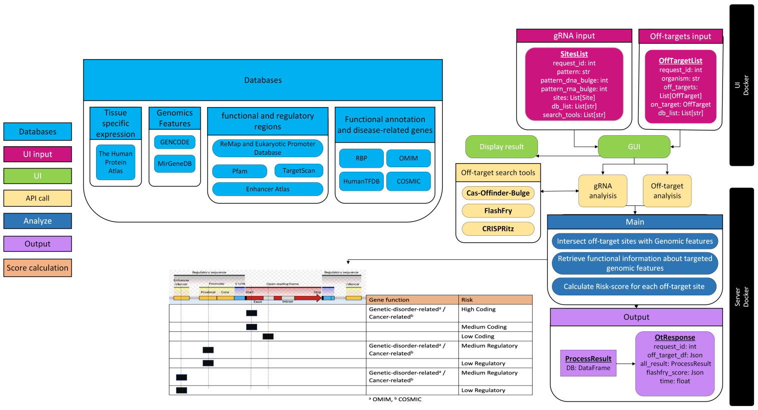

Figure 1: OffRisk complete structure.

The UI docker is written with Python model streamlit [1]. Two modes of input are currently available:

1. Off-targets input: the input is a list of off-target sites; each one is represented by its genomic location: chromosome, start, end, and strand This input can be supplied on the off-target page in a text box or as a tsv file. The list of

sites has to be in a tab delimiter format, where each site is given in a different row. An example of a site location: [chr1 88873884 88873910 +].

2. gRNA input: the input is a gRNA sequence with PAM. Additional information about the upstream and downstream sequences can be added for search off-targets with FlashFry (see below). In the input box, one gRNA sequence can be supplied at a time. For more than one gRNA, a JSON file can be used. The gRNA input is used to find all off-target sites. The user can choose between Cas-OFFinder [2], FlashFry [3] or CRISPRitz [4] for this task. For FlashFry the upstream and downstream sequences are mandatory.

The off-target sites, that are provided in the first mode or found in the second mode, are forwarded to the main analysis step as described for the Server docker below. For both modes, the user can select on which databases to run the analysis and select the server to work on - local or public.

1.1.1 Web UI docker

The UI docker allows users to create requests for the server docker and present the final results that include tables and graphs. The following is a description of the steps performed: (1) The user selects on which page to run the analysis (on-target or off-target); (2) The user selects relevant databases; (3) The user enters the requested input and clicks on "Run"; (4) Once the request from the server returns, results for each database are displayed on the web UI results page.

1.1.2 Server docker

The server docker performs the main analysis, which aims to bring biological information for the off-target sites, with the two following steps: 1. Intersecting the genomic locations of the off-target sites with the locations of the genomic features (e.g., GENCODE, miRGeneDB) using BedTools intersect [5]. 2. Extracting for the genomic features identified in step 1 additional information from other databases (using a common identifier, e.g., Ensembl ID for GENCODE). 3. Finally, the results are returned in JSON format with the structure of OtResponse as seen in Figure 1. The full structure of OtResponse can be found in additional material.

1.2 Technical considerations

OffRisk was built using docker infrastructure and therefore can be deployed on Windows and Linux operating systems that support Docker Engine (https://www.docker.com/). A tutorial is available from the GitHub repository.

The code is written in python 3.7.9. Additional scripts are written in python as well and are available on the docker and on git. These scripts are used to pre-process the databases (see section Materials and Methods).

2 Materials and method

2.1 Databases

Our pipeline currently supports off-target evaluation for the human genome (version GRCh38 [6]). To analyze the biological context of a given set of off-target sites, we integrated various resources, as described below. These resources include information about genomic coding and non-coding features, e.g., their location in the genome and functional data (Table 1). Briefly, ”BedTools intersect” [5] is used to identify genomic features that overlap with off-target sites using genomic locations provided in resources, e.g., GENCODE, MirGeneDB, ReMap with EPD, Enhancer Atlas, Pfam, and TargetScan. Then, resources, e.g., OMIM, HumanTFDB, The Human Protein Atlas, resource of RNA-binding proteins, and COSMIC, are used to retrieve functional annotations of these features using an identifier id that is common to these resources (e.g., Ensembl ID).

Below is the description of the integrated resources, the type of information they provide, and the **pre-processing** steps that were applied to extract only relevant information into convenient file formats, which take place only once during setup. The pre-processing code can be found in the python script *preprocess.py* as part of the docker server and on GitHub.

In addition, we describe the **analysis** steps that are applied during the application. Each database is processed and prepared for display in the UI in dedicated tables and the final summary table for each off-target site.

Table 1: Supported Databases

Database name	Description	Database version	Genome version	link
GENCODE	Gene features in human based on biological evidence	Release v42	GRCh38.p13, GRCh38/hg38	GENCODE
MirGeneDB	Database of microRNA genes that have been validated and annotated	Version 2.1	GRCh38/hg38	MirGeneDB
ReMap and Eukaryotic Promoter Database	ReMap is a database of transcriptional regulators from DNA-binding experiments. EPD is a database of transcription initiation sites of eukaryotic genes' promoters.	ReMap: release 4 2022, EPD: coding - version 6, non-coding - version 1	ReMap and EPD: Dec 2013 GRCh38/hg38, GENCODE v28	ReMap, EPD
Enhancer Atlas	Enhancers database, distal cis-regulatory elements that activate the transcription of their target genes	Version 2.0	GRCh37/hg19	Enahncer Atlas
Pfam	Database of protein families and domains		GRCh38/hg38	Pfam
TargetScan	Database for predicted microRNA targets in mammals	8.0	GRCh37/hg19	TargetScan

OMIM	A comprehensive, authoritative compendium of human genes and genetic phenotypes	Downloaded on December 24, 2020		File were provided by request
HumanTFDB	Database with information on animal transcription factors (TFs) and cofactors	Version 3.0		HumanTFDB
Published resource of RNA-binding proteins	Data set of RNA elements in the human genome that are recognized by RNA-binding proteins (RBPs)			RBP
COSMIC	Database for the available information about the effects of somatic mutations across the range of human cancers	Release version 96, 31st May 2022		COSMIC
The Human Protein Atlas	Database which shows the distribution of the proteins across all major tissues and organs in the human body	Version 21.1		Human Protein Atlas

All database files except OMIM and COSMIC are publicly available and are provided as part of the dockers. OMIM and COSMIC require licenses; therefore, users who want to use them need to download and apply a pre-processing step as described in the manual.

2.1.1 Resources for genomics features

GENCODE: GENCODE [7] project aims to identify and classify all gene features in the human and mouse genomes with high accuracy based on biological evidence, and to release these annotations for the benefit of biomedical research and genome interpretation. We downloaded *gencode.v42.chr_patch_hapl_scaff-.annotation.gff3* from `https://www.gencodegenes.org/human/` (Comprehensive gene annotation, ALL regions) on November 2022.

Pre-processing: (1) Change the names of the *chromosome* to be in the same format as NCBI; (2) remove records related to pseudogenes; (3) sort the file according to *chromosome*, *start* and *end* position for better performance using python *pybedtools* package; (4) save the file as *gencode.v40.chr_patc_hapl_scaff-.annotation_sort.gff3*.

Analysis: Run *pybedtools intersect* to match off-target sites with GENCODE records. Aggregate rows according to the *off-target ID* and *gene Ensembl ID*. Then the aggregated records are examined by the *segment (feature)* field. If present, only "CDS", "five_prime_UTR" or "three_prime_UTR" records are kept. Otherwise, the most specific type of feature record is kept - "exon", "transcript", or "gene". The summary table shows the GENCODE hits that overlap off-target sites (columns *gene Ensembl ID*, *gene symbol*, and *segment*).

MirGeneDB: MirGeneDB [8] is a public database of bonafide miRNA genes. Version 2.0 contains more than 10,000 genes from 45 organisms represent-

ing nearly every major metazoan group, and these microRNAs can be browsed, searched, and downloaded. We downloaded from `https://mirgenedb.org/download` human miRNAs in a BED file, downloaded on November 2022.

Pre-processing: (1) Change the names of the *chromosome* to be in NCBI format; (2) sort the file according to *chromosome*, *start* and *end* positions for better performance; (3) save the file as *mirgene.bed*.

Analysis: Run *pybedtools intersect* to match off-target sites with MirGeneDB records. Aggregate rows according to the *miRNA name*, and keep only one row for each name (the first one intersecting with the off-target site). The summary table shows the MirGeneDB hits that overlap off-target sites (column *miRNA symbols*).

2.1.2 Resources for functional and regulatory regions

ReMap and Eukaryotic Promoter Database ReMap [9] aims to provide manually curated, high-quality catalogs of regulatory regions resulting from a large-scale integrative analysis of DNA-binding experiments in Human, Mouse, Fly, and Arabidopsis thaliana for hundreds of transcription factors and regulators. The Human regulatory atlas includes 8103 datasets covering 1210 transcriptional regulators (TRs) with a catalog of 182 million (M) peaks. We downloaded from `http://remap.univ-amu.fr/download_page` the H. sapiens BED file for the non-redundant peak on November 2022.

Eukaryotic Promoter Database (EPD) [10]is a promoter resource whose primary purpose is to keep track of experimental data that define transcription initiation sites of eukaryotic genes. This type of functional information is linked to promoter sequences via machine-readable pointers to positions within sequences of the EMBL nucleotide sequence database. We downloaded from `https://epd.epfl.ch/EPDnew_select.php` H. sapiens promoters for coding and non-coding genes in BED format on November 2022.

Pre-processing: For EPD only (1) for each file, replace *symbol* with relevant *Ensembl ID*; (2) merge the files with a flag indicating if this record is from the coding region or not - 1 for coding, 0 for non-coding. Then, for both EPD and ReMap files (3) merge these two files using *Bedtools intersect* to contain the information on the peak of the DNA binding factor and the promoter of the gene it belongs to; (4) change the names of the *chromosome* to be in NCBI format; (5) save the file as *remap_epd.bed*.

Analysis: Run *pybedtools intersect* to match off-target sites with TF binding sites on promoter regions. The summary table shows *gene Ensembl IDs* of genes whose promoters overlap off-target sites *Promoter of Gene (ENSG)*.

Enhancer Atlas: EnhancerAtlas [11] provides enhancer annotation in several species, including human. Enhancers are distal cis-regulatory elements that activate the transcription of their target genes. They regulate a wide range of important biological functions and processes, including embryogenesis, development, and homeostasis. We have downloaded from `http://www.enhanceratlas.org/downloadv2.php` all enhancer-gene interactions for human on November 2022.

Pre-processing: (1) Combine all enhancer-gene interactions to one BED file, and add *tissue/cell types* name to each record; (2) Convert coordinates from hg19 to hg38 (GRCh38) using LiftOver command line tool [12]; (3) change the names of the chromosome to NCBI format; (4) save the file as *enhancerAtlas.bed*.

Analysis: Run *pybedtools intersect* to match off-target sites with regions of enhancer-gene interaction. The summary table shows *gene Ensembl ID* of genes whose enhancer region overlaps an off-target site.

Pfam protein domains: Pfam [13] is a database of protein families and domains that is widely used to analyze novel genomes and metagenomes, and to guide experimental work on particular proteins and systems. Each Pfam family has a seed alignment that contains a representative set of sequences for the entry. A profile hidden Markov model (HMM) is automatically built from the seed alignment and searched against a sequence database called pfamseq using the HMMER software (http://hmmer.org/). All sequence regions that satisfy a family-specific curated threshold, also known as the gathering threshold, are aligned to the profile HMM to create the full alignment. It is worth noting that a common misuse of Pfam is to use a single E-value threshold across all Pfam HMMs, which results in lower sensitivity and an increase in false positive matches when compared to using the per-family gathering thresholds. Pfam entries are manually annotated with functional information from the literature where available. We Downloaded from https://genome.ucsc.edu Pfam data for human assembly GRCH38 on November 2022.

Pre-processing: (1) Separate each location into different rows; (2) convert *chromosome* names to NCBI format; (3) add the *gene Ensembl Id* information from GENCODE; (4) save the file as *pfam_protein_domains.bed*.

Analysis: Run *pybedtools intersect* to match off-target sites with Pfam domains. The summary table shows all *pfam domain names* that overlap off-target sites.

TargetScan: TargetScan [14] predicts biological targets of miRNAs by searching for the presence of conserved 8mer, 7mer, and 6mer sites that match the seed region of each miRNA. Also identified are sites with mismatches in the seed region that are compensated by conserved 3' pairing. In mammals, predictions are ranked based on the predicted efficacy of targeting as calculated using a biochemical model of miRNA-mediated repression, which was extended to all miRNA sequences using a convolutional neural network. As an option, predictions are also ranked on the basis of targeting efficacy estimated using cumulative weighted context++ scores of the sites. As another option, predictions are ranked by their probability of conserved targeting. TargetScanHuman considers matches to human 3' UTRs and their orthologs, as defined by UCSC whole-genome alignments. Conserved targeting has also been detected within open reading frames (ORFs). We downloaded from `https://www.targetscan.org/cgi-bin/targetscan/data_download.vert80.cgi`, version 8.0 the file "Genome coordinates of Predicted Conserved Targets" in hg19 coordinates.

Pre-processing: (1) Convert the coordinates to hg38 using LiftOVer; (2) save the file as *targetscan.bed*.

Analysis: Run *pybedtools intersect* to match the off-target sites with miRNA-

target sites. The summary table shows all *miRNA symbols* whose target sites overlap off-target sites.

2.1.3 Resources for Functional annotation and disease-related genes

OMIM: Online Mendelian Inheritance in Man (OMIM) [15]is a comprehensive, authoritative compendium of human genes and genetic phenotypes that is freely available and updated daily. The full-text, referenced overviews in OMIM contain information on all known mendelian disorders and over 15,000 genes. We requested access to the following files from the site `https://www.omim.org/contact`: (1) mim2gene.txt: a tab-delimited file linking MIM numbers with NCBI Gene IDs, Ensembl Gene IDs, and HGNC Approved Gene Symbols; and (2) genemap2.txt: a tab-delimited file containing OMIM's Synopsis of the Human Gene Map including additional information such as genomic coordinates and inheritance. We received access to the data on December 2020.

Pre-processing: (1) Merge both files based on their *omim id*; (2) keep the columns with the following information: *Chromosome, Genomic Position Start, Genomic Position End, MIM Number, Phenotypes, Approved Gene Symbol (HGNC) and Ensembl Gene ID (Ensembl)*; (3) separate the information on the *inheritance model* (column Phenotype) into a different column; (4) save the file as *omim.csv*.

Analysis: Intersect OMIM records with GENCODE, ReMap_EPD and Enhancer Atlas using *gene Ensembl ID*, into separated tables. The intersection results for GENCODE are shown in a table, while the results for ReMap_EPD and Enhancer Atlas are used to calculate the *risk score* (see below). The summary table shows *phenotype* information for GENCODE hits, ReMap_EPD, and Enhancer Atlas in separate columns.

HumanTFDB: HumanTFDB [16] is a resource aimed to provide information for animal transcription factors (TFs) and cofactors. We downloaded from `http://bioinfo.life.hust.edu.cn/HumanTFDB/#!/download` two files: Human TF as _TF.txt and Human TF Cofactor as Homo_sapiens_TF_cofactors.txt on November 2022.

Pre-processing: (1) Merge the two tab-delimited files into one file, and add a column *source* to indicate the source file - "TF" or "TF Cofactor"; (2) Save the file as *human_tf.bed*.

Analysis: Intersect HumanTF records with GENCODE. The intersection results are shown in a table. The summary table displays the column *HumanTF source* indicating if GENCODE hit is TF/TF cofactor.

Published resource of RNA-binding proteins (RBP): A publication by Van Nostrand et al [17] introduced a new data set of manual annotation of RBP functions. We downloaded the file from `https://www.nature.com/articles/s41586-020-2077-3#MOESM3` in section Supplementary information the file "Data summary and manual annotation of RBP functions" as *41586_2020_2077_MOESM3_ESM.xlsx* on November 2022.

Pre-processing: (1) Extract columns from *Essential Genes* to *Other*; (2) save the file as *rbp.csv*.

Analysis: Intersect RBP records with GENCODE. The intersection results are presented as a heatmap to show gene function.

COSMIC: The Catalogue Of Somatic Mutations In Cancer (COSMIC) [18], draws together the available information about the effects of somatic mutations across the range of human cancers. The primary data in COSMIC are derived directly from the scientific literature by expert manual curators, who read and digest journal articles and extract the detailed mutation data within, along with any additional information such as environmental factors or patient pre-disposition that may be accessible. In parallel, a second curation tracks data from systematic screens, via the major cancer data portals and from the supplementary tables and downloadable files associated with curated papers. Data from these two curation strands is combined to give COSMIC an unrivaled breadth and depth of coverage, making it the primary resource for the exploration of the etiology and landscape of mutations in human cancer. We Downloaded from `https://cancer.sanger.ac.uk/cosmic/download` the file "Cancer Gene Census" on November 2022.

Pre-processing: (1) Keep the information on *Synonyms, gene_symbol, Name, Somatic, Germline, Tumour Types(Somatic), Tumour Types(Germline), Molecular Genetics and Role in Cancer*; (2) filter all records that did not have any information on Role in cancer; (3) saved the file as *cosmic.csv*.

Analysis: Intersect *gene Ensembl ID* extracted from GENCODE hits, ReMap_EPD and Enhancer Atlas hits, with COSMIC data, into separated tables. The result from GENCOCE are shown as a table to the user, and the results from ReMap_EPD and Enhancer Atlas are used to calculate the score. The summary table displays *role in cancer* for relevant records from GENCODE, ReMap_EPD and Enhancer Atlas, each in a separate column.

2.1.4 Resources for Tissue-specific expression

The Human Protein Atlas The Human Protein Atlas[19] is a Swedish-based program initiated in 2003 with the aim to map all the human proteins in cells, tissues, and organs using an integration of various omics technologies. The Human Protein Atlas consists of six separate parts, each focusing on a particular aspect of the genome-wide analysis of human proteins. We used only the Tissue Atlas, which shows the distribution of the proteins across all major tissues and organs in the human body. We downloaded from `https://www.proteinatlas.org/about/download` normal_tissue.tsv.zip on November 2022.

Pre-processing: (1) Count the number of occurrences for each *tissue-cell type* combination and filter all counts that are below 50; (2) Write for all gene Ensembl IDs their expression levels under tissue/cell type and save in *protein_atlas.csv* file.

Analysis: (1) Intersect *gene Ensembl ID* extracted from GENCODE hits with Ensembl IDs from the atlas; (2) Map the expression values to 6 options – *Not representative (0), None (1), Not detected (2), Low (3), Medium (4), High (5)*; (3) Present the results as heatmap according to the expression value. The summary table displays a vector of expression levels for relevant records from

GENCODE.

2.1.5 Risk-Score calculation

We calculate a **risk score** for each off-target site based on the genomic feature it hits and the associated function of the feature as can be seen in 2: (1) "High Coding": off-target overlaps with an exon of a protein-coding gene, and the gene has a genetic-disorder-related or cancer-related function. (2) "Medium coding": off-target overlaps with an exon of a protein-coding gene, and there is no evidence for genetic-disorder-related or cancer-related function. (3) "Low Coding": off-target overlaps with non-exonic regions of a protein-coding gene. (4) "Medium Regulatory": off-target overlaps with promoter or enhancer region of a gene that has a genetic-disorder-related or cancer-related function. (5) "Low Regulatory": off-target overlaps with promoter or enhancer region of a gene, and there is no evidence for genetic-disorder-related or cancer-related function. All other cases will have no score.

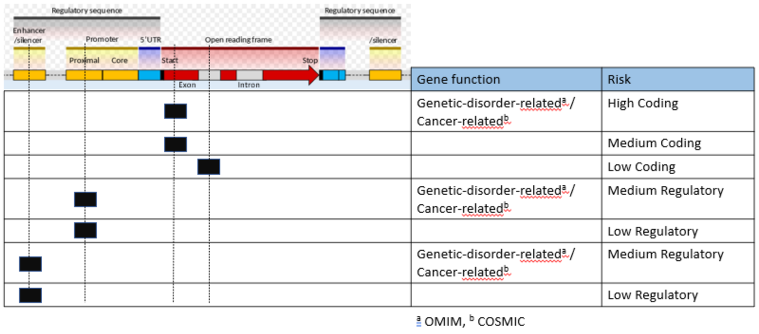

Figure 2: Score calculation

2.2 External packages

We used the following external packages in our pipeline.

2.2.1 BedTools

BedTools is a command-line tool for the comparison, manipulation, and annotation of genomic features in Browser Extensible Data (BED) and General Feature Format (GFF) format. The tools are extremely efficient and allow the user to compare large datasets (e.g., next-generation sequencing data) with both public and custom genome annotation tracks [5]. We used pybedtools, a python wrapper that offers feature-level manipulations from Python [20] to intersect between the off-target sites to databases that provide genomic coordinates, e.g., GENCODE, MirGeneDB, ReMap and EPD, Enahncer Atlas, Pfam, and TargetScan.

2.2.2 RCircos

RCircos is an R package, implementing Circos [21]. The package supports Circos 2D data track plots such as scatter, line, histogram, heatmap, tile, connectors, links, and text labels. Each plot is implemented with a specific function. Input data for all functions is supplied as a data frame that can be read from text files or generated with other R pipelines [22]. We use RCircos to visualize in a circular graph the locations of the off-targets on the genome.

2.2.3 Streamlit

Streamlit is an open-source Python library that makes it easy to create and share custom web apps for machine learning and data science [1].

We use the following tools to search for off-targets for given guide sequences in the "gRNA input" mode: FlashFry [3], Cas-Offinder [2], and CRISPRitz [4]. In case FlashFry is invoked, results also include a table with FlashFry scores for the input gRNA. Cas-OFFinder-bulge is a wrapper that can handle RNA/DNA bulges, we use a similar idea to support search with bulges in both Cas-OFFinder and CRISPRitz. FlashFry is limited by searching only NGG PAM and gRNA of size 20 nts. We extract from the output of all tools, the genomic locations of the off-target sites, the guide and target sequences, and the number of mismatches between the guide and the target site.

References

[1] Arvindra Sehmi, Ashish Shukla, Ceyda Cinarel, Charly Wargnier, Christian Klose, Christina Frezynski, Fanilo Andrianasolo, Jesse Agbemabiase, Johannes Rieke, José Manuel Nápoles, Robin Cole, Dorian Oukil, and Tyler Richards. *streamlit*, July 2021.

[2] Sangsu Bae, Jeongbin Park, and Jin-Soo Kim. Cas-OFFinder: a fast and versatile algorithm that searches for potential off-target sites of Cas9 RNA-guided endonucleases. *Bioinformatics*, 30(10):1473–1475, 01 2014.

[3] Aaron McKenna and Jay Shendure. FlashFry: a fast and flexible tool for large-scale CRISPR target design. *BMC Biology*, 16(1), July 2018.

[4] Samuele Cancellieri, Matthew C Canver, Nicola Bombieri, Rosalba Giugno, and Luca Pinello. Crispritz: rapid, high-throughput and variant-aware in silico off-target site identification for crispr genome editing. *Bioinformatics*, 36(7):2001–2008, 2020.

[5] Aaron R. Quinlan and Ira M. Hall. BEDTools: a flexible suite of utilities for comparing genomic features. *Bioinformatics*, 26(6):841–842, 01 2010.

[6] U.S. National Library of Medicine National Center for Biotechnology Information. Grch38, 2021.

[7] Adam Frankish, Mark Diekhans, Anne-Maud Ferreira, Rory Johnson, Irwin Jungreis, Jane Loveland, Jonathan M Mudge, Cristina Sisu, James Wright, Joel Armstrong, If Barnes, Andrew Berry, Alexandra Bignell, Silvia Carbonell Sala, Jacqueline Chrast, Fiona Cunningham, Tomás Di Domenico, Sarah Donaldson, Ian T Fiddes, Carlos García Girón, Jose Manuel Gonzalez, Tiago Grego, Matthew Hardy, Thibaut Hourlier, Toby Hunt, Osagie G Izuogu, Julien Lagarde, Fergal J Martin, Laura Martínez, Shamika Mohanan, Paul Muir, Fabio C P Navarro, Anne Parker, Baikang Pei, Fernando Pozo, Magali Ruffier, Bianca M Schmitt, Eloise Stapleton, Marie-Marthe Suner, Irina Sycheva, Barbara Uszczynska-Ratajczak, Jinuri Xu, Andrew Yates, Daniel Zerbino, Yan Zhang, Bronwen Aken, Jyoti S Choudhary, Mark Gerstein, Roderic Guigó, Tim J P Hubbard, Manolis Kellis, Benedict Paten, Alexandre Reymond, Michael L Tress, and Paul Flicek. GENCODE reference annotation for the human and mouse genomes. *Nucleic Acids Research*, 47(D1):D766–D773, October 2018.

[8] Bastian Fromm, Diana Domanska, Eirik Høye, Vladimir Ovchinnikov, Wenjing Kang, Ernesto Aparicio-Puerta, Morten Johansen, Kjersti Flatmark, Anthony Mathelier, Eivind Hovig, Michael Hackenberg, Marc R Friedländer, and Kevin J Peterson. MirGeneDB 2.0: the metazoan microRNA complement. *Nucleic Acids Research*, 48(D1):D132–D141, 10 2019.

[9] Fayrouz Hammal, Pierre de Langen, Aurélie Bergon, Fabrice Lopez, and Benoit Ballester. ReMap 2022: a database of Human, Mouse, Drosophila and Arabidopsis regulatory regions from an integrative analysis of DNA-binding sequencing experiments. *Nucleic Acids Research*, 50(D1):D316–D325, 11 2021.

[10] Rouaïda Cavin Périer, Viviane Praz, Thomas Junier, Claude Bonnard, and Philipp Bucher. The Eukaryotic Promoter Database (EPD). *Nucleic Acids Research*, 28(1):302–303, 01 2000.

[11] Tianshun Gao and Jiang Qian. EnhancerAtlas 2.0: an updated resource with enhancer annotation in 586 tissue/cell types across nine species. *Nucleic Acids Research*, 48(D1):D58–D64, 11 2019.

[12] Robert M. Kuhn, David Haussler, and W. James Kent. The UCSC genome browser and associated tools. *Briefings in Bioinformatics*, 14(2):144–161, 08 2012.

[13] Jaina Mistry, Sara Chuguransky, Lowri Williams, Matloob Qureshi, Gustavo A Salazar, Erik L L Sonnhammer, Silvio C E Tosatto, Lisanna Paladin, Shriya Raj, Lorna J Richardson, Robert D Finn, and Alex Bateman. Pfam: The protein families database in 2021. *Nucleic Acids Research*, 49(D1):D412–D419, 10 2020.

[14] Vikram Agarwal, George W Bell, Jin-Wu Nam, and David P Bartel. Predicting effective microrna target sites in mammalian mrnas. *eLife*, 4:e05005, aug 2015.

[15] MD) McKusick-Nathans Institute of Genetic Medicine, Johns Hopkins University (Baltimore. Online mendelian inheritance in man, omim®.

[16] Hui Hu, Ya-Ru Miao, Long-Hao Jia, Qing-Yang Yu, Qiong Zhang, and An-Yuan Guo. AnimalTFDB 3.0: a comprehensive resource for annotation and prediction of animal transcription factors. *Nucleic Acids Research*, 47(D1):D33–D38, 09 2018.

[17] Eric L. Van Nostrand, Peter Freese, Gabriel A. Pratt, Xiaofeng Wang, Xintao Wei, Rui Xiao, Steven M. Blue, Jia-Yu Chen, Neal A. L. Cody, Daniel Dominguez, Sara Olson, Balaji Sundararaman, Lijun Zhan, Cassandra Bazile, Louis Philip Benoit Bouvrette, Julie Bergalet, Michael O. Duff, Keri E. Garcia, Chelsea Gelboin-Burkhart, Myles Hochman, Nicole J. Lambert, Hairi Li, Michael P. McGurk, Thai B. Nguyen, Tsultrim Palden, Ines Rabano, Shashank Sathe, Rebecca Stanton, Amanda Su, Ruth Wang, Brian A. Yee, Bing Zhou, Ashley L. Louie, Stefan Aigner, Xiang-Dong Fu, Eric Lécuyer, Christopher B. Burge, Brenton R. Graveley, and Gene W. Yeo. A large-scale binding and functional map of human rna-binding proteins. *Nature*, 583(7818):711–719, Jul 2020.

[18] John G Tate, Sally Bamford, Harry C Jubb, Zbyslaw Sondka, David M Beare, Nidhi Bindal, Harry Boutselakis, Charlotte G Cole, Celestino Creatore, Elisabeth Dawson, Peter Fish, Bhavana Harsha, Charlie Hathaway, Steve C Jupe, Chai Yin Kok, Kate Noble, Laura Ponting, Christopher C Ramshaw, Claire E Rye, Helen E Speedy, Ray Stefancsik, Sam L Thompson, Shicai Wang, Sari Ward, Peter J Campbell, and Simon A Forbes. COSMIC: the Catalogue Of Somatic Mutations In Cancer. *Nucleic Acids Research*, 47(D1):D941–D947, 10 2018.

[19] Mathias Uhlén, Linn Fagerberg, Björn M. Hallström, Cecilia Lindskog, Per Oksvold, Adil Mardinoglu, Åsa Sivertsson, Caroline Kampf, Evelina Sjöstedt, Anna Asplund, IngMarie Olsson, Karolina Edlund, Emma Lundberg, Sanjay Navani, Cristina Al-Khalili Szigyarto, Jacob Odeberg, Dijana Djureinovic, Jenny Ottosson Takanen, Sophia Hober, Tove Alm, Per-Henrik Edqvist, Holger Berling, Hanna Tegel, Jan Mulder, Johan Rockberg, Peter Nilsson, Jochen M. Schwenk, Marica Hamsten, Kalle von Feilitzen, Mattias Forsberg, Lukas Persson, Fredric Johansson, Martin Zwahlen, Gunnar von Heijne, Jens Nielsen, and Fredrik Pontén. Tissue-based map of the human proteome. *Science*, 347(6220), 2015.

[20] Ryan K. Dale, Brent S. Pedersen, and Aaron R. Quinlan. Pybedtools: a flexible Python library for manipulating genomic datasets and annotations. *Bioinformatics*, 27(24):3423–3424, 09 2011.

[21] Martin I Krzywinski, Jacqueline E Schein, Inanc Birol, Joseph Connors, Randy Gascoyne, Doug Horsman, Steven J Jones, and Marco A Marra. Circos: An information aesthetic for comparative genomics. *Genome Research*, 2009.

[22] Hongen Zhang, Paul Meltzer, and Sean Davis. RCircos: an r package for circos 2d track plots. *BMC Bioinformatics*, 14(1):244, 2013.
